# Group A *Streptococcus* Is Still at Large

**DOI:** 10.3390/jcm12072739

**Published:** 2023-04-06

**Authors:** Qinghua Lu, Dingle Yu, Yonghong Yang

**Affiliations:** 1 Microbiology Laboratory, Beijing Pediatric Research Institute, Beijing Children’s Hospital, Capital Medical University, National Center for Children’s Health, Beijing 100045, China; 2 Respiratory Department, Shenzhen Children’s Hospital, Shenzhen 518038, China

Group A *Streptococcus* (GAS) is a major human pathogen that can cause diseases, ranging from mild to severe systemic and invasive diseases. It has a major impact on global mortality and morbidity. Recent data from the UK show an unusually high incidence of severe GAS, an increase in the reported cases of scarlet fever, and the development of invasive group A streptococcal disease (iGAS). Unfortunately, iGAS has been responsible for the deaths of children in the UK over the past few months [1,2]. Meanwhile, the severity and number of iGAS cases in Spain and France increased significantly in 2022, compared to the pre-pandemic COVID-19 period [3,4].

The factors associated with the outbreak of GAS infections abroad are analyzed as follows. First, it may be due to the fact that during the COVID-19 pandemic, affected children were in confinement, leading to increased susceptibility of children to the pathogen-—the so-called “immune debt” [5]. That is, the non-seasonal nature of the current case outbreak is most likely due to the increase in children who are not yet infected with GAS. Reduced exposure to GAS during the COVID-19 blockade has led to a lack of immunity to GAS in some children, slowing the natural build-up of immunity levels in children and leading to the emergence of a greater proportion of susceptible children [6]. After the relaxation of COVID-19 restrictions, many pathogens caused non-seasonal outbreaks, especially in children, often at higher rates than before the COVID-19 pandemic [7]. Younger children are the most affected, probably because of their delayed first exposure to these pathogens, but older children and adults have also lost the opportunity to acquire enhanced natural immunity that was previously acquired through seasonal exposure. In addition, early GAS infection may be easily mistaken for SARS-CoV-2 Omicron infection by parents and even health care workers, because it also has GAS pharyngitis-like symptoms (fever and sore throat), which may easily delay appropriate antibiotic treatment. Secondly, changes in bacterial genome and virulence may make GAS more susceptible, such as the increase in GAS infections in 2014 coinciding with the increase in iGAS infections and the emergence of major virulent GAS lineages [8]. In the past decade, there was a sudden resurgence of scarlet fever in several countries and regions, which has attracted the attention of scholars all over the world. The 2017–2018 outbreak of GAS infection was considered to be caused by a sublineage of GAS strains containing high levels of the scarlet fever toxin SpeA that appeared in the UK in 2016 [9]. The results of routine laboratory surveillance of antimicrobial susceptibility in the UK showed no increase in antibiotic resistance in circulating GAS strains. Therefore, there is no evidence that new strains are spreading and ongoing surveillance is still needed. Compared to the period when the pandemic control measures were implemented, the elevated iGAS cases in children are likely to be a consequence of the heightened scarlet fever activity, given the crossover of strains associated with both presentations [10]. It is important to clarify the cause of the scarlet fever “resurgence”. When social distancing finally eases, scarlet fever is likely to make a comeback. Thirdly, asymptomatic carriers can cryptogenically transmit the disease [11]. Therefore, it is also important to determine whether asymptomatic patients or carriers of the virus can undergo mixing (or secondary) as is the case for those with active viral infections. More research in this area is urgently needed to gain insight into the current situation. In addition, reports of seasonal influenza and COVID-19 may play an important role [1]. Infection with iGAS is associated with the co-transmission of some winter viruses, such as respiratory syncytial virus and influenza, because infection with one pathogen may increase the risk of a second pathogen, and the virus breaks the respiratory barrier so that GAS can enter more easily. So, GAS and most respiratory viruses have the same seasonal pattern of infection rates and they are likely to interact to influence the outcome of the infection. The severity of respiratory infections significantly increases when bacterial infections occur simultaneously (i.e., co-infection) or after viral infections (i.e., secondary infections) [12]. Since the 1918 influenza pandemic, it has been shown that bacterial infections can be complicated by viral influenza infections, and the most common cause of severe and fatal bronchopneumonia is secondary influenza A infection with *Streptococcus pneumoniae* or GAS, and both influenza A and influenza B viruses are associated with an increased risk of severe GAS infection [13]. Although there are few data on mixed infections of influenza A virus and GAS, the available data suggest that the severity of mixed infection is higher than that of single infections [14].

We agree that the most plausible theory that could explain the high incidence of iGAS in children in UK is that respiratory viral infections may be associated with iGAS; therefore, seasonal influenza and COVID-19 may also have a very important role [3]. It is also possible that the exposure to Streptococci is reduced during the COVID-19 blockade, resulting in a lack of immunity to GAS in some children [1].

In the UK, UK officials advocate the use of antibiotics as prophylactic drugs to stop the epidemic. We disagree with this view because it runs counter to the Global Action Plan on Antibiotics and Bacterial Resistance. For example, in China, skin testing is required before penicillin can be prescribed, which limits the use of penicillin. As a result, the use of macrolides is very high in China, and the GAS for resistance to macrolides is also very high. The high resistance rates and high levels of clindamycin and macrolides have hindered their use as alternatives to GAS in China. Although, no GAS isolate with resistance to β-lactam antibiotics has emerged so far. However, *pbp2x* gene variants have been reported to cause an increase in the minimum inhibitory concentration (MIC) values of GAS to penicillin, which requires vigilance for the emergence of truly β-lactam antibiotics-resistant GAS strains [15]. The “immune debt” people owe to bacterial infections such as GAS cannot be ignored in the early stages of strict control of the COVID-19 pandemic. When the preventive and control measures are relaxed, GAS infections are likely to occur. Children with significant sore throats after infection should be especially vigilant.

We advocate rapid diagnosis by antigen detection, which is important for the rational use of antibiotics for early (bedside) diagnosis of GAS infection, rather than prophylactic use of antimicrobials to combat the GAS epidemic. The rapid diagnosis of GAS infection and early application of antibiotics can reduce clinical symptoms, avoid complications, and reduce transmission rates. The rapid antigen detection test (RADT) is the most commonly used method for the rapid diagnosis of GAS by directly detecting its group specific antigens from throat swabs using immuno-detection methods, such as latex agglutination or colloidal gold, which can provide an important reference for early, real-time clinical treatment. Due to its low sensitivity, if RADT is negative, the specimen needs to be retested by culture or PCR. Random-access, integrated molecular devices available at the point of care will facilitate the rapid and accurate diagnosis of GAS infections, leading to prompt treatment.

There are some unsolved conundrums in the control of GAS infections. Firstly, in the last decade, there was a sudden “resurgence” of scarlet fever in several countries, and the cause is not entirely clear. Secondly, the reasons for the persistent in vitro susceptibility of GAS to β-lactams are not known. However, β-lactam treatment failures in the clinical setting have been documented over decades, and for reasons that are not fully understood. Reduced-penicillin-susceptibility GAS has been reported in recent years. The emergence of the PBP mutation is also worrying [15]. Thirdly, in China, skin tests are required before penicillin is used, which limits its use to some extent.

The increased and more severe GAS scarlet fever cases being noticed in the UK have attracted our attention, especially in the face of a sharp change in China’s COVID-19 policy. Both GAS pharyngeal tonsillitis and SARS-CoV-2 Omicron infection cause fever with a sore throat, so they are easily misdiagnosed. In the context of the global pandemic of COVID-19, the rapid diagnosis and prompt treatment for single and co-infections of GAS and SARS-CoV-2 are recommended to reduce the risk of possible complications and limit onward transmission. Clinicians should continue to pay attention to the potential increase in invasive cases, remain highly suspicious of relevant patients, and provide appropriate safety recommendations, as the early identification of patients infected with iGAS and timely initiation of specific and supportive treatment can save lives. Despite the difficulties, it is hoped that an effective GAS vaccine will be available soon.
